# Creating the Coupled Band Gaps in Piezoelectric Composite Plates by Interconnected Electric Impedance

**DOI:** 10.3390/ma11091656

**Published:** 2018-09-07

**Authors:** Lin Li, Zhou Jiang, Yu Fan, Jun Li

**Affiliations:** 1 School of Energy and Power Engineering, Beihang University, Beijing 100191, China; feililin@buaa.edu.cn (L.L.); buaa_jiangzhou@buaa.edu.cn (Z.J.); 2 Beijing Key Laboratory of Aero-Engine Structure and Strength, Beijing 100191, China; 3 Collaborative Innovation Center for Advanced Aero-Engine, Beijing 100191, China; 4 Department of Aeronautics, Imperial College London, London SW7 2AZ, UK; jun.li16@imperial.ac.uk

**Keywords:** piezoelectric composite plates, interconnected electric network, locking phenomenon, coupled band gap, wave and finite element method

## Abstract

In this paper, we investigate the coupled band gaps created by the locking phenomenon between the electric and flexural waves in piezoelectric composite plates. To do that, the distributed piezoelectric materials should be interconnected via a ‘global’ electric network rather than the respective ‘local’ impedance. Once the uncoupled electric wave has the same wavelength and opposite group velocity as the uncoupled flexural wave, the desired coupled band gap emerges. The Wave Finite Element Method (WFEM) is used to investigate the evolution of the coupled band gap with respect to propagation direction and electric parameters. Further, the bandwidth and directionality of the coupled band gap are compared with the LR and Bragg gaps. An indicator termed ratio of single wave (RSW) is proposed to determine the effective band gap for a given deformation (electric, flexural, etc.). The features of the coupled band gap are validated by a forced response analysis. We show that the coupled band gap, despite directional, can be much wider than the LR gap with the same overall inductance. This might lead to an alternative to adaptively create band gaps.

## 1. Introduction

Periodic structures feature frequency band gaps (also termed the *stop bands*) in which certain wave mode (Bloch wave modal shape) cannot propagate and the associated energy flow is forbidden [1,2,3]. Such unique wave filtering characteristics can find applications in vibration reduction [4,5,6], energy focus [7] and even acoustic cloaking [8]. There are three main mechanisms to create a band gap, namely the Bragg scattering, local resonance (LR) and locking phenomena respectively.

The creation of a Bragg or a LR band gap attributes to the frequency evolution of a single wave mode, as the results of interference generated by the interaction of incident and scattered waves at the unit-cell boundaries that periodically presented in space [9]. The Bragg band gaps appear around frequencies governed by the Bragg condition L=n(λ/2) where *n* is an integer, λ is the wavelength and *L* is the unit-cell length (also termed the lattice constant). This implies that the structural periodicity must be of the same order as the wavelength of the band-gap frequencies, as observed in periodic engineering structures such as truss beams [10], perforated plates [11], and stiffened cylinders [12]. If the impedance mismatch in the unit cell involves an internal resonance, then a band gap centred at the resonant frequency can be created, termed the local-resonance (LR) band gap [13]. Thus, the LR band gaps are not restricted to wavelengths on the same order as the lattice spacing, and they can lie in the sub-wavelength regime whereby waves with wavelengths larger than that of the unit cell will be prohibited from propagation. Please note that the creation of Bragg and LR band gaps does not necessarily need two different configurations of the unit cell. Transition between the Bragg and LR band gaps can occur once the parameters of the same kind of periodicity satisfy certain criteria [14].

However, a coupled band gap is created by the frequency evolution and interaction of two *weakly coupled* wave modes [15,16,17], notably when the *locking* phenomenon between such two waves is triggered. The term ‘couple’ does not mean that the two wave modes break the orthogonal relations but refers to the fact that they comprise more than one basic form of wave (termed the ‘uncoupled’ waves). Typical examples are the torsional-bending waves in a beam with an asymmetric cross section [18,19]. The term ‘weak’ means a relatively lower magnitude of the coupling force compared with the magnitudes of the inertial and elastic forces in the uncoupled waveguides [16]. This could also mean that the coupling coefficient is relatively low compared with the mass and stiffness coefficients. In this regard, the locking phenomenon will be triggered once: (1) the dispersion curves of the uncoupled waves intersect, namely the waves have identical wavelength at the intersection frequency; and (2) the uncoupled waves have opposite group velocity when they are approaching to intersect. Consequently, an identical band gap for each coupled wave will appear around the intersection frequency, and it is referred to as the coupled band gap. Please note that if the signs of the group velocity for the uncoupled waves are the same, the *veering* phenomenon will be triggered and the coupled band gaps will not be created. General physical and mathematical descriptions of the locking phenomenon in elastic waves can be found in the work of Mace and Manconi [16].

**Figure 1:**
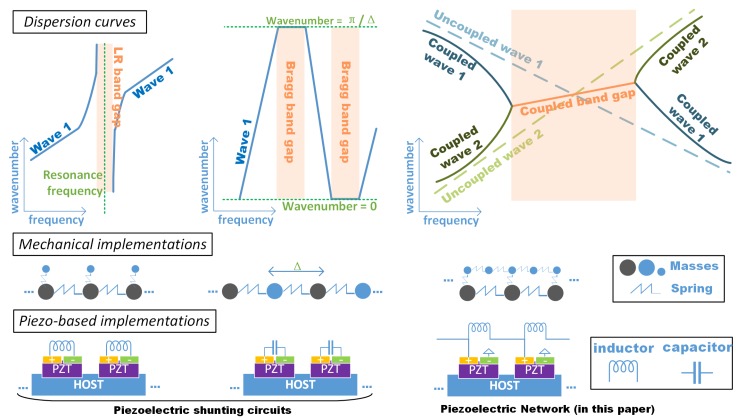
Schematic illustration of the main features for the dispersion curves, the mechanical and piezoelectric-based implementations for the Bragg, LR and coupled band gaps.

The main features of the Bragg, LR and coupled band gaps are illustrated in Figure 1. The creation of the Bragg and LR band gaps explicitly requires the presence of periodicity as mentioned. As for the coupled band gaps, one of the uncoupled waves should have a declining dispersion curve, so as to satisfy the requirement of ‘opposite group velocity’. This cannot be the case of a uniform structure, but can be achieved by the presence of periodicity where the waves in the propagating zone between two Bragg band gaps may have such features [18,19].

The engineering of periodic structures involves the creation of the band gaps in the desired frequency zone. Due to periodicity, the wave propagation characteristics of a periodic structure are governed by the dynamics of a single unit cell (smallest repetitive substructure). Hence one can intentionally design the material and geometric parameters, as well as the boundary conditions of the unit cell to tailor or artificially create band gaps [20,21]. These artificial periodic structures are often referred to as *phononic crystals* [5,22]. Especially, the periodic structures with LR band gaps have been regarded as a new kind of *acoustic metamaterials* for they possess novel (effective) physical behaviour such as negative mass and/or stiffness [7,23]. Pure mechanical implementations of the band gaps require high machining precision and cannot adjust the features in accordance with the environmental changes. Alternatively, if we introduce piezoelectric materials to the unit cell, and deploy local electric impedance (via the shunting circuits) to the electrodes as shown in Figure 1; the electric impedance will be transmitted to the mechanical field and equivalent to for example local stiffness or mass [24,25] thanks to the piezoelectric effects. This provides light-weight and adaptive implementations for the Bragg [6,26] and LR band gaps [27,28,29,30].

Engineering the coupled band gaps can be a difficult task for both mechanical and piezoelectric-shunting implementations. Both two waves for the creation of a coupled band gap can be tailored by the change of material and geometric parameters, or via the local electric impedance, therefore triggering the locking phenomenon at the desired frequency can be a more challenging task than the creation of a Bragg or a LR band gap. This issue impedes the utilization of the locking phenomenon in the design of structures with extensive band gaps.

**Figure 2:**
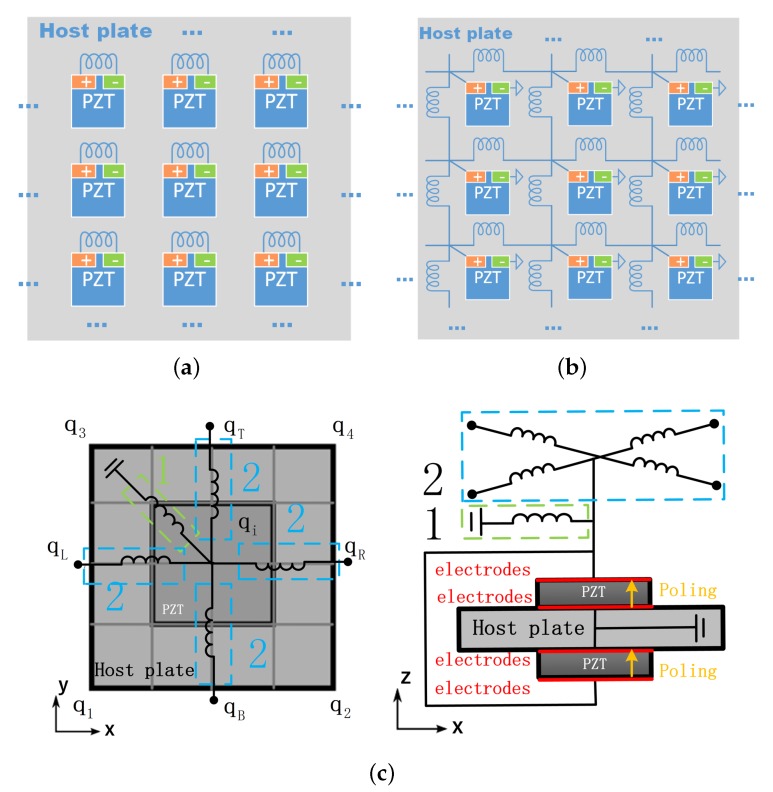
Piezoelectric composite plates with (**a**) local shunting impedance and (**b**) one form of the global electric impedance (considered in this paper). The unit cell of the piezoelectric composite plates is shown in (**c**), where ‘1’ and ‘2’ refer to shunting and network circuits respectively.

In this paper, we conduct theoretical and numerical analysis, adaptively creating the coupled band gaps by interconnecting the electrodes in a piezoelectric composite plate via an inductive electric network, as shown in Figure 2b. This interconnected configuration of piezoelectric system is termed the ‘piezoelectric network’ in the literature [31,32] and also in this paper. An example of the 1D case is illustrated in Figure 1 as well. Please note that the network connection can have many forms, as proposed by Maurini et al. [33]. The intrinsic capacitance of the piezoelectric materials and the interconnected inductance as a whole performs like a mass-spring network, and can be regarded as an attached ‘elastic media’ to the host structure. This leads to an additional electromechanical wave [31,32,33,34,35,36,37]. Yi et al. [34] used this wave to trigger the veering phenomenon to modify the out-of-plane wave in a piezoelectric composite plate, such that acoustic coincidence frequency is cancelled and a very good sound isolation performance is achieved. Alessandroni et al. [35] tailored the created electromechanical wave such that it has the same dispersion relation with the target wave in the host plate, leading to a multi-mode vibration absorber. Yu and Wang [31], and Liu et al. [32] utilized the created electromechanical wave as an additional energy transmission path, to destroy the localization mechanism in the near-periodic structures. Despite that the additional electromechanical wave is promising to trigger the locking phenomenon with the existing mechanical waves, to the authors’ knowledge, no previous work has been devoted to the detailed analysis of the creation and the characteristics of coupled band gaps by means of a piezoelectric network.

A uniform plate is considered as the host structure, for it is representative for 2-dimensional waveguides and it underlies many engineering applications. Piezoelectric materials (PZT-5H) are periodically bonded to the plate, and in each unit cell two piezoelectric patches are collocated and their electrodes are connected such that the electromechanical coupling only exists for the flexural waves (Figure 2). A simple electric network is imposed, comprising only inductance as shown in Figure 2b. Theoretical solutions for the dispersion curves in such an electric network is available, which can be used to predict the creation of coupled band gaps. Please note that a LR band gap can be created, if we shunt an identical inductance to the piezoelectric materials in each unit cell, as shown in Figure 2a. This motivates us to also compare the features of the LR and coupled band gaps by the same amount of overall inductance. The results can lead to an alternative mean to create band gaps in piezoelectric composites by inductance, and can advance the related fields such as vibration control and sound isolation.

In the remainder of this paper, we will introduce the numerical tools for the analysis of the band gaps in the piezoelectric composite plates (Section 2); investigate the characteristics of uncoupled (Section 3.1) and coupled (Section 3.2) waves; compare the coupled band gaps with the Bragg band gaps (Section 3.3) and LR band gaps (Section 3.4); and validate the obtained results by forced response analysis (Section 4).

## 2. Wave and Finite Element Method (WFEM)

Since it is difficult to find analytical solutions for the considered electromechanical plates, in this paper a numerical tool termed the wave finite element method (WFEM) is adopted to analyse the wave propagation characteristics in the piezoelectric composite plates. WFEM requires only the modelling of a unit cell for the analysis of time harmonic wave characteristics and forced response of periodic structures [10,19,38,39,40], therefore it is much faster than the conventional finite element method. It can be applied to piezoelectric structures with external electric circuits [41,42,43] once the finite element model of such an electromechanical unit cell is obtained and the degree-of-freedoms (DOFs) are partitioned appropriately. The dispersion curves of the waves are obtained by solving the eigenvalue problem which can be formulated in many different ways [44].

In this work we use ANSYS 17 (ANSYS, Inc., Canonsburg, PA, USA) to model the host plate and the piezoelectric materials. The *HBMAT* command in ANSYS is used to export the mass, stiffness and damping matrices to the external files with the Harwell-Boeing Format. The Matlab *HB_TO_MSM* program is used to recover these sparse matrices from the external files into Matlab workspace, and the *full* Matlab function is used to convert those data to full matrices. An in-house Matlab code is developed to give the finite element model of the electric circuits. The mechanical and electric models of the unit cell are assembled in the Matlab code, yielding a complete model of the unit cell. All the following-up procedures of WFEM, including the solving of the eigenvalue problem, and the post-processing are implemented by an in-house Matlab code developed by the authors. For the sake of clarity, we briefly outline the method in this section.

The unit cells of the two kinds of piezoelectric composite shown in Figure 2 can be represented in a uniform way as shown in Figure 2c. The dynamic equations of the unit cell write:

(1) $$M\ddot{q}+C\dot{q}+Kq=f$$

where q is the generalized coordinates vector containing both mechanical and electric DOFs. Please note that in piezoelectric theory the full electromagnetic equations are not usually needed. The quasi-electrostatic approximation is adequate because the phase velocities of acoustic waves are approximately five orders of magnitude less than the velocities of electromagnetic waves. Under these circumstances magnetic effects can be shown to be negligible compared with electric effects [45]. Thus in this paper we use the term *electric* instead of *electromagnetic* to describe the corresponding variables, forces, waves etc. The mechanical DOFs consist of the nodal displacement while the electric DOFs refer to the nodal flux (whose first derivative with time is nodal voltage). Likewise, f is the generalized force vector which contains both force and charge loads. In this regard, M, C and K are generalized mass, damping and stiffness matrices respectively. Choosing the magnetic flux as nodal DOF enables us to incorporate inductance, resistance and capacitance into K, C and M respectively [46].

With regard to harmonic motions, the dynamic equations of a unit cell at frequency ω are given by

(2) $$\left[\begin{array}{cc} {\hat{D}}_{\mathrm{ii}} & {\hat{D}}_{\mathrm{ib}}\\ {\hat{D}}_{\mathrm{bi}} & {\hat{D}}_{\mathrm{bb}} \end{array}\right]\left(\begin{array}{c} q_{i}\\ q_{b} \end{array}\right)=\left(\begin{array}{c} f_{i}=0\\ f_{b} \end{array}\right)$$

where $\hat{D}=K+j\omega C-\omega^{2}M$ is the dynamic stiffness matrix (DSM). Please note that the vectors and matrices in Equation (1) are partitioned according to the boundary DOFs (with subscript ‘b’) and internal DOFs (with subscript ‘i’). In case of free wave propagation, there is no external load applied at the internal DOFs, i.e., $f_{i}=0$. Hence the internal DOFs $q_{i}$ can be condensed, leading to:

(3) $$Dq_{b}=f_{b}$$

where

(4) $$D={\hat{D}}_{\mathrm{bb}}-{\hat{D}}_{\mathrm{bi}}{\hat{D}}_{\mathrm{ii}}^{-1}{\hat{D}}_{\mathrm{ib}}$$

is the condensed DSM.

Next, we partition vectors of $q_{b}$ and $f_{b}$ as: four corners $q_{1}$, $q_{2}$, $q_{3}$ and $q_{4}$; left $q_{L}$; bottom $q_{B}$; right $q_{R}$, top $q_{T}$. These notations are illustrated also in Figure 2c as well. Note that when global impedance is applied, there are electric DOFs both in the boundary and internal vectors, namely in $q_{L}$, $q_{R}$, $q_{T}$, $q_{B}$ and $q_{i}$. Only the internal vector $q_{i}$ has electric DOFs if only the local impedance is applied. Accordingly the nodal displacement and force vectors can now be expressed as:

(5) $$q_{b}={\left[\begin{array}{cccccccc} q_{1} & q_{2} & q_{3} & q_{4} & q_{L} & q_{B} & q_{R} & q_{T} \end{array}\right]}^{T}$$

and

(6) $$f_{b}=[\begin{array}{cccccccc} f_{1} & f_{2} & f_{3} & f_{4} & f_{L} & f_{B} & f_{R} & f_{T} \end{array}{]}^{T}$$

According to periodic structure theory [47], the aforementioned nodal displacement vector has the following relation:

(7) $$q_{b}=T\left(\lambda_{x},\lambda_{y}\right)\hat{q}$$

where

(8) $$\hat{q}={\left[\begin{array}{ccc} q_{1} & q_{L} & q_{B} \end{array}\right]}^{T}$$

Similarly, due to the periodicity and the equilibrium of the internal force, we have

(9) $$T^{T}\left({\lambda_{x}}^{-1},{\lambda_{y}}^{-1}\right)f_{b}=0$$

where the matrix T writes

(10) $$T=\left[\begin{array}{ccc} I & 0 & 0\\ \lambda_{x}I & 0 & 0\\ \lambda_{y}I & 0 & 0\\ \lambda_{x}\lambda_{y}I & 0 & 0\\ 0 & I & 0\\ 0 & 0 & I\\ 0 & \lambda_{x}I & 0\\ 0 & 0 & \lambda_{y}I \end{array}\right]$$

In these equations, $\lambda_{x}=e^{\mu_{x}}$ and $\lambda_{y}=e^{\mu_{y}}$, where $\mu_{x}=-jk_{x}L_{x}$ and $\mu_{y}=-jk_{y}L_{y}$ are the propagation constants respectively along *x* and *y* directions; $j=\sqrt{-1}$; $k_{x}$ and $k_{y}$ are wavenumber with which the wave propagates along *x* and *y* directions; $L_{x}$ and $L_{y}$ are the length of unit cell along the *x* and *y* directions.

Substituting Equations (7) and (9) into Equation (4), we have:

(11) $$T^{T}\left({\lambda_{x}}^{-1},{\lambda_{y}}^{-1}\right)D\left(\omega \right)T\left(\lambda_{x},\lambda_{y}\right)\hat{q}=0$$

where three parameters ω, $\lambda_{x}$ and $\lambda_{y}$ are unknown. In this paper, we solve Equation (11) by fixing one of the propagation constants (say $\lambda_{y}$) and the frequency ω, leading to a quadratic eigenvalue problem in the other propagating constant ($\lambda_{x}$):

(12) $$\left(\lambda_{x}X+Y+\lambda_{x}^{-1}Z\right)\hat{q}=0$$

which can be solved by the *polyeig* function in Matlab. The detailed formulas of matrices **X**, **Y** and **Z** can be found in Appendix A. Please note that it is also feasible to solve the eigenvalue problem by other schemes such as having $k_{x}$ to be real and reaching for complex ω, to get the so called ‘phase constant surface’ and in this case the ω with an imaginary part indicates an evanescent wave [10,48]. It can be an alternative to analyse the wave propagations characteristics but the carried information and conclusions will be the same as the currently employed numerical scheme.

Each solution of Equation (12) represents a plane wave of shape $\hat{q}$ travels freely in the structure at frequency ω with wavenumber $k_{x}$ and $k_{y}$. Repeating this calculation at different frequencies, dispersion curves $\left(k_{x},\omega \right)$ for the fixed $k_{y}$ can be obtained. Sometimes it is more intuitive to present the angular wave number $k_{\theta }=\sqrt{k_{x}^{2}+k_{y}^{2}}$ and its evolution along $\theta =\arctan(k_{y}/k_{x})$, and they will be used later to illustrate the directionality of wave propagation.

Given the dispersion curves (k,ω) where *k* can be either $k_{x}$ or $k_{y}$, the characteristics of the wave can be known by the properties of *k*. First let us consider the undamped case, there are three situations:

1. If *k* is a real number, namely λ is a complex number whose amplitude is 1, this indicates a propagating wave. The wave is propagating along the positive direction of the axis if k>0, along the negative direction if k<0.
2. If *k* is a complex number with real(k) equals to nπ where n is an integer, namely λ is a real number less (positive going) or larger (negative going) than 1, this indicates an evanescent wave. There is no phase change during the passage of this kind of evanescent wave, which is the case for the waves in a Bragg or a LR band gap.
3. If *k* is a complex number but real(k) does not equal to nπ, namely λ is a complex number whose amplitude is less (positive going) or larger (negative going) than 1, this also indicates an evanescent wave. However there are both decaying of the amplitude and phase change associated with this wave, which is the case for the waves in a coupled band gap.

The presence of damping mechanisms, including the materials damping and resistance in the circuits, may turn the propagating waves into evanescent ones (case 3). Then all the waves will be attenuated during passage. The damped waveguides can also be analysed by the WFEM formulas described in this paper through the introduction of damping matrix C in Equation (1). It is worth to note that the attenuation caused by damping is associated by the energy dissipation, but the attenuation caused by a band gap is related to energy reflection [41]. Thus, band gap and damping are two different mechanisms to achieve wave isolation. For this reason, in this paper we concentrate on the analysis of the coupled band gaps and do not consider any dissipation or damping.

**Figure 3:**
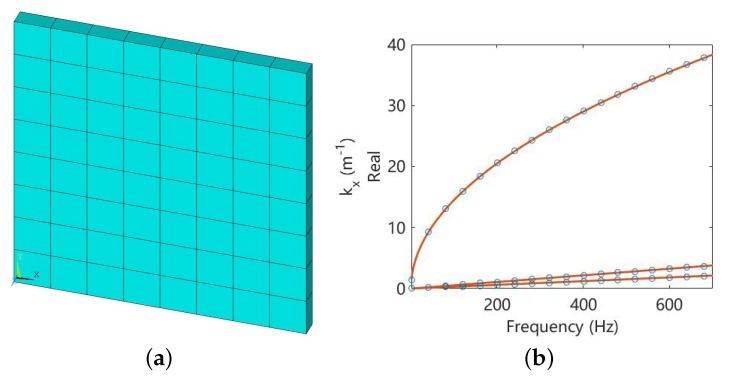
Unit cell model (**a**) and (**b**) the dispersion curves of the propagation waves long the *x* direction in the uniform host plate. The theoretical solutions are denoted by ∘ and the WFEM results are denoted by −. These are three curves, and they represent the longitudinal, torsional and flexural waves respectively. Note that in (**a**), the thickness of the shell element is shown.

The WFEM code is first benchmarked against the uniform host plate, as shown in Figure 3. The analytical solutions for wavenumber of the longitudinal ($k_{l}$), shear ($k_{s}$) and flexural waves ($k_{f}$) at angular frequency ω are:

(13) $$k_{l}=\omega /\sqrt{\frac{E}{(1-\nu^{2})\rho }}$$

(14) $$k_{s}=\omega /\sqrt{\frac{E}{2(1+\nu )\rho }}$$

(15) $$k_{f}^{2}=\omega /\sqrt{\frac{Eh^{2}}{12(1-\nu^{2})\rho }}$$

respectively, where Young’s modulus $E=4.35\times 10^{9}\;\mathrm{Pa}$, Poisson’s ratio ν=0.37, mass density $\rho =1.18\times 10^{3}\;kg/m^{3}$ and thickness $h=5\times 10^{-3}\;m$. In WFEM, the plate unit cell (Figure 3a) is modelled by SHELL181 element in ANSYS, which is a four-node element with six degrees of freedom at each node. The comparison is presented in Figure 3b, where the WFEM results match very well with the theoretical solutions. This also verifies that the mesh density as well as the choice of element type are correct. Another benchmark of the WFEM code is presented in the next section.

## 3. Results and Discussions

The considered host plate is made of epoxy with Young’s modulus 4.35 N/$m^{2}$, Poisson ratio 0.37 and mass density $1.18\times 10^{3}\;{\mathrm{kg}/m}^{3}$. The piezoelectric materials PZT-5H (see Appendix B for the material parameters) are collocated and the size of each PZT patch is $40\times 40\times 0.2\;mm^{3}$. The unit cell size for such a piezoelectric composite is $80\times 80\times 5\;mm^{3}$. Later, the plate with interconnected inductance is referred to as ‘L-network’ and the plate with locally shunted inductance is referred to as ‘L-shunt’.

### 3.1. The Uncoupled Waves

As mentioned, the creation of a coupled band depends on the properties of the uncoupled waves. In this section, we analyse the mechanical and electric waves in their respective uncoupled media. There are two ways to define the uncoupled systems [16], namely the *uncoupled disconnected* system and the *uncoupled blocked* system. In the uncoupled disconnected system the coupled forces are removed, while in the uncoupled blocked system the displacement of the other wave is forced to be zero. In the case of the considered piezoelectric composite plate, let us rewrite Equation (1) by partitioning q into the mechanical $q_{M}$ and electric $q_{E}$ vectors, written:

(16) $$\left[\begin{array}{cc} {\hat{D}}_{\mathrm{MM}} & {\hat{D}}_{\mathrm{ME}}\\ {\hat{D}}_{\mathrm{EM}} & {\hat{D}}_{\mathrm{EE}} \end{array}\right]\left(\begin{array}{c} q_{M}\\ q_{E} \end{array}\right)=\left(\begin{array}{c} f_{M}\\ f_{E} \end{array}\right)$$

By definition, the uncoupled disconnected mechanical system is obtained by setting the coupled forces to zero, namely ${\hat{D}}_{\mathrm{EM}}q_{M}+{\hat{D}}_{\mathrm{EE}}q_{E}=0$, leading to:

(17) $$\left({\hat{D}}_{\mathrm{MM}}-{\hat{D}}_{\mathrm{ME}}{\hat{D}}_{\mathrm{EE}}^{-1}{\hat{D}}_{\mathrm{EM}}\right)q_{M}=f_{M}$$

Likewise, the uncoupled disconnected electric system is obtained by setting the coupled forces to zero, ${\hat{D}}_{\mathrm{MM}}q_{M}+{\hat{D}}_{\mathrm{ME}}q_{E}=0$:

(18) $$\left({\hat{D}}_{\mathrm{EE}}-{\hat{D}}_{\mathrm{EM}}{\hat{D}}_{\mathrm{MM}}^{-1}{\hat{D}}_{\mathrm{ME}}\right)q_{E}=f_{E}$$

On the other hand, the unit cell of the uncoupled blocked mechanical system is governed by:

(19) $${\hat{D}}_{\mathrm{MM}}q_{M}=f_{M}$$

and the uncoupled blocked electric system is governed by:

(20) $${\hat{D}}_{\mathrm{EE}}q_{E}=f_{E}$$

It can be seen that the uncoupled disconnected systems refer to the mechanical waveguide with the *open circuit* electric boundary condition and the electric waveguide with the *force free* mechanical boundary condition. The uncoupled blocked systems refer to the mechanical waveguide with the *short circuit* electric boundary condition and the electric waveguide with all the mechanical displacement constrained. Starting from Equations (17)–(20), and following the aforementioned WFEM procedures, the wave propagation characteristics of these uncoupled systems can be obtained.

**Figure 4:**
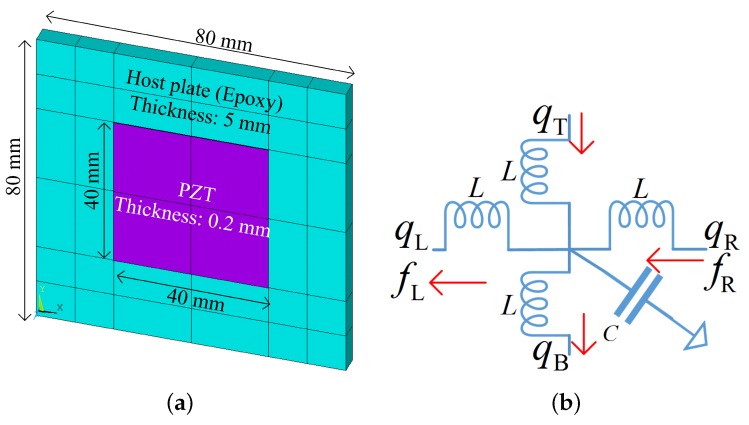
The unit cells of the uncoupled mechanical (**a**) and electric (**b**) systems. Note that the thickness of the shell elements is illustrated in (**a**).

**Figure 5:**
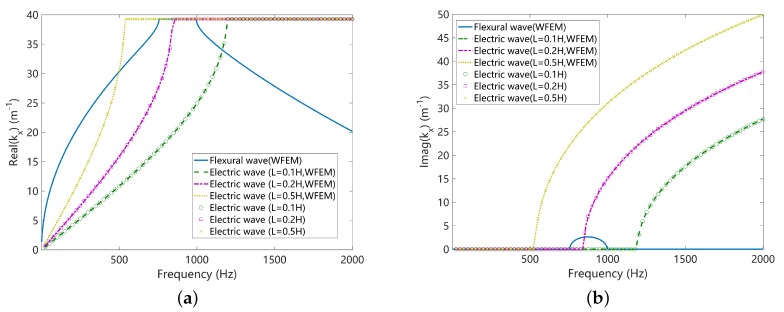
The dispersion curves of the uncoupled disconnected systems: (**a**) real part of the wavenumber; (**b**) imaginary part of the wavenumber. The numerical results obtained by WFEM are validated against the theoretical solutions enclosed in Appendix C for the electric waves.

The unit cells of the uncoupled mechanical and electric systems are shown in Figure 4 where the host plate is modelled by the SHELL181 elements in ANSYS and the piezoelectric patches by the SOLID5 elements. The wave propagation characteristics of uncoupled disconnected systems along the x direction are analysed, by setting $\lambda_{y}=1$ and searching for $\lambda_{x}$ at each frequency ω. The results are shown in Figure 5, where only the flexural wave of the uncoupled mechanical system is presented. The Bragg band gap of the mechanical wave is induced by the periodicity associated by the presence of piezoelectric materials. Please note that there is only one propagating zone for the electric wave because of the configuration of the considered circuit form. After this propagating zone there is a permanent Bragg band gap. The slope of the electric waves can be controlled by the inductance. We highlight three circumstances where the uncoupled electric waves will intersect with the uncoupled mechanical wave (flexural wave):

1. When L=0.1H, the uncoupled waves intersect with opposite sign of group velocity. Thus the requirements for the creation of a coupled band gap are satisfied. This case will be further studied in the next section to verify this and to study the properties of the created coupled band gap.
2. When L=0.2H, the uncoupled electric wave intersects with a Bragg band gap of the uncoupled mechanical wave, where the group velocity of the mechanical wave is zero. Whether a coupled band gap will appear in this case is not clearly stated in the literature. We will further study this case in the next section to clarify this point.
3. When L=0.5H, the uncoupled waves intersect with the same sign of group velocity. The veering phenomenon will be triggered and no coupled band gap will appear by expectation. This case will be further studied in the next section to verify this and to study the electromechanical properties induced by the veering phenomenon.

In this paper we use the uncoupled disconnect waves as references to understand the coupled waves, this will be justified in the next section. Theoretical solutions are available (enclosed in Appendix C) for the uncoupled disconnect electric waves, and they are plotted in Figure 5. Good agreements can be observed, justifying the correctness of the WFEM code.

### 3.2. Band Gap Structure and Its Identification of the Coupled Systems

**Figure 6:**
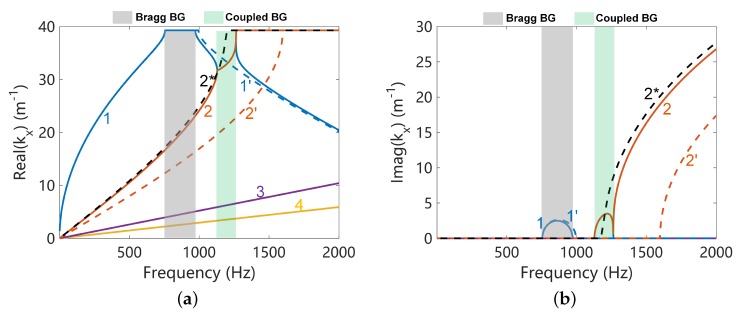
Dispersion curves along the *x* direction of L-network with L=0.1H, (**a**) shows the real part of $k_{x}$ and (**b**) shows the imaginary part of $k_{x}$. Waves 1 and 2 are electromechanical waves. The dash lines 1’ and 2* refer to uncoupled disconnected flexural and electric waves respectively. The dash line 2’ refers to the uncoupled blocked electric wave.

The dispersion curves along *x* direction of L-network with L=0.1H are shown in Figure 6. Four propagation waves are observed, including two electromechanical waves, shear wave and longitudinal wave. Two Bragg band gaps are observed when waves 1 and 2 satisfying $k_{x}/L_{x}=\pi$ where $L_{x}$ is the unit cell width along *x* direction. The intersection frequency of the uncoupled disconnected flexural (1’) and electric (2*) waves has good agreement with the coupled band gap, as shown in Figure 6. However the intersection frequency for the uncoupled blocked electric wave (2’) and mechanical wave (does not presented because it is very close to 1’) is about 200 Hz higher than the coupled band gap. Moreover, the uncoupled blocked electric wave even does not agree with the coupled electric wave (2) at frequencies much lower than the intersection zone. Thus in our case the uncoupled blocked waves are not appropriate references and we use the uncoupled disconnected waves as references for the understanding of the coupled band gaps.

**Figure 7:**
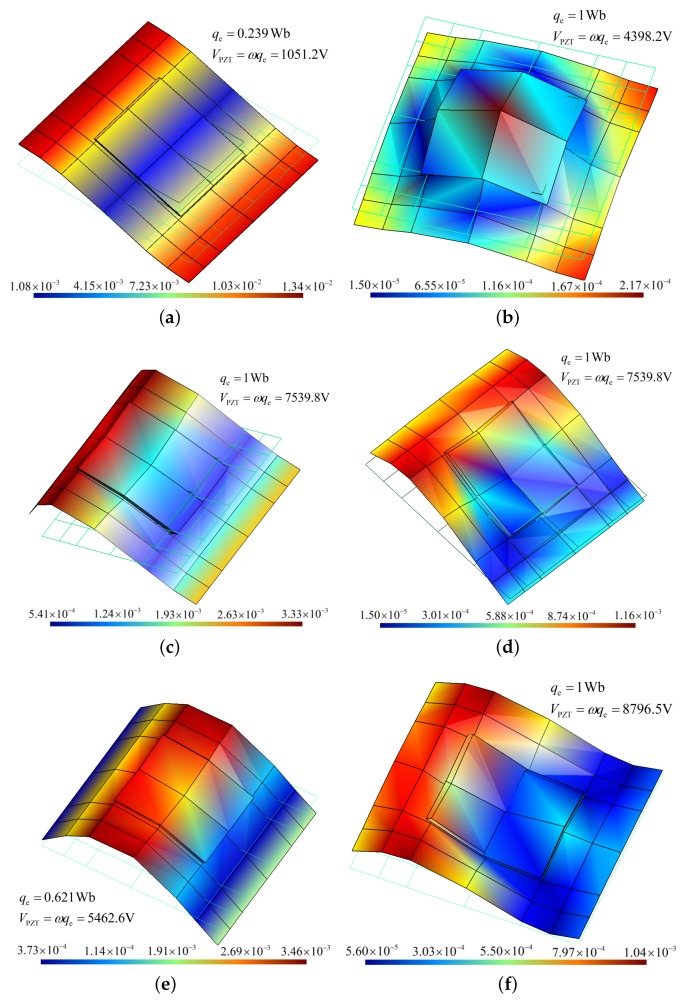
Waveshapes of: (**a**) wave 1 at 700 Hz; (**b**) wave 2 at 700 Hz; (B) wave 1 at 1200 Hz; (**d**) wave 2 at 1200 Hz; (**e**) wave 1 at 1400 Hz; (**f**) wave 2 at 1400 Hz. The MAC (modal assurance criterion) between (**a**) and (**b**) is 0.0097, MAC between (**c**) and (**d**) is 0.8420, MAC between (**e**) and (**f**) is 0.2048. The waveshapes are normalized such that the amplitude of the largest DOFs is 1.

In the coupled band gap, waves 1 and 2 have exactly the same wavenumber, and their waveshapes are very similar as shown in Figure 7c,d. We also find that at frequencies far below the coupled band gap, wave 1 is dominated by the flexural deformation and wave 2 by electric field. Their waveshapes are no longer similar as shown in Figure 7a,b. This also happens at frequencies above the coupled band gap as shown in Figure 7e,f. All these features confirm that such a coupled band gap is corresponding to the locking phenomenon of waves 1 and 2.

As mentioned, wave 2 is dominated by electric field for frequencies above the locking zone (the coupled band gap), therefore the Bragg band gap of wave 2 can not be used to block the energy transmission through the flexural deformation. This is also because that at the same frequencies wave 2 is a propagating wave and can be excited by the external forces that generate flexural deformation. This leads us to the problem of identifying the ‘effective’ band gap for one specific deformation. This can be a trivial question sometimes. For example, it is easy to find that none of the presented band gaps in Figure 6 are ‘effective’ for pure shear and longitudinal deformations. Because we know easily that the waves 1 and 2 don’t have any shear and longitudinal component. However, for waves with complex shape that contains several ‘basic’ deformations, e.g., the torsional-flexural waves in an open thin-wall structure, identifying the ‘effective’ band gap for the pure torsional deformation is not an easy task.

In this paper, the question is how to identify the ‘effective’ band gap for the flexural and electric deformation. For this we propose the following indicator termed the Ratio of Single Wave (RSW):

(21) $$\mathrm{RSW}=\mathrm{MAC}\left(\phi_{\mathrm{ref}},\phi_{\mathrm{full}}\right)=\frac{{\left|\phi_{\mathrm{ref}}^{H}\;\cdot \;\phi_{\mathrm{full}}\right|}^{2}}{\left(\phi_{\mathrm{ref}}^{H}\;\cdot \;\phi_{\mathrm{ref}}\right)\left(\phi_{\mathrm{full}}^{H}\;\cdot \;\phi_{\mathrm{full}}\right)}$$

where $\phi_{\mathrm{full}}$ is the actual wave shape of the system and $\phi_{\mathrm{ref}}$ is the reference wave shape representing the target deformation. For example in our case the shell elements are used to construct the FE model of the unit cell, so $\phi_{\mathrm{full}}={\left[u_{x},u_{y},u_{z},\theta_{x},\theta_{y},\theta_{z},\lambda \right]}^{T}$. If we would like to know how much flexural deformation this wave mode $\phi_{\mathrm{full}}$ has, then we let

(22) $$\phi_{\mathrm{ref}}=\phi_{f}={\left[0,0,u_{z},\theta_{x},\theta_{y},0,0\right]}^{T}$$

and introduce it into Equation (21) to get ${\mathrm{RSW}}_{f}=\mathrm{RSW}\left(\phi_{f}\right)$. RSW is a real value in [0,1], where 0 means $\phi_{\mathrm{full}}$ does not contain any contribution of deformation $\phi_{\mathrm{ref}}$ and 1 means $\phi_{\mathrm{full}}$ is fully contributed by $\phi_{\mathrm{ref}}$. In this work, if RSW<0.1, we consider no contribution of $\phi_{\mathrm{ref}}$ in $\phi_{\mathrm{all}}$. Further, if all the propagation waves in the given frequency band are satisfying RSW<0.1 with $\phi_{\mathrm{ref}}$, we consider this frequency band as the band gap of the deformation $\phi_{\mathrm{ref}}$.

Similarly, we can define ${\mathrm{RSW}}_{e}=\mathrm{RSW}\left(\phi_{e}\right)$ for the electric deformation where $\phi_{e}={\left[0,0,0,0,0,0,\lambda \right]}^{T}$, ${\mathrm{RSW}}_{s}=\mathrm{RSW}\left(\phi_{s}\right)$ for the shear deformation where $\phi_{s}={\left[0,0,0,0,0,\theta_{z},0\right]}^{T}$, and ${\mathrm{RSW}}_{l}=\mathrm{RSW}\left(\phi_{l}\right)$ for the longitudinal deformation where $\phi_{l}={\left[u_{x},u_{y},0,0,0,0,0\right]}^{T}$.

**Figure 8:**
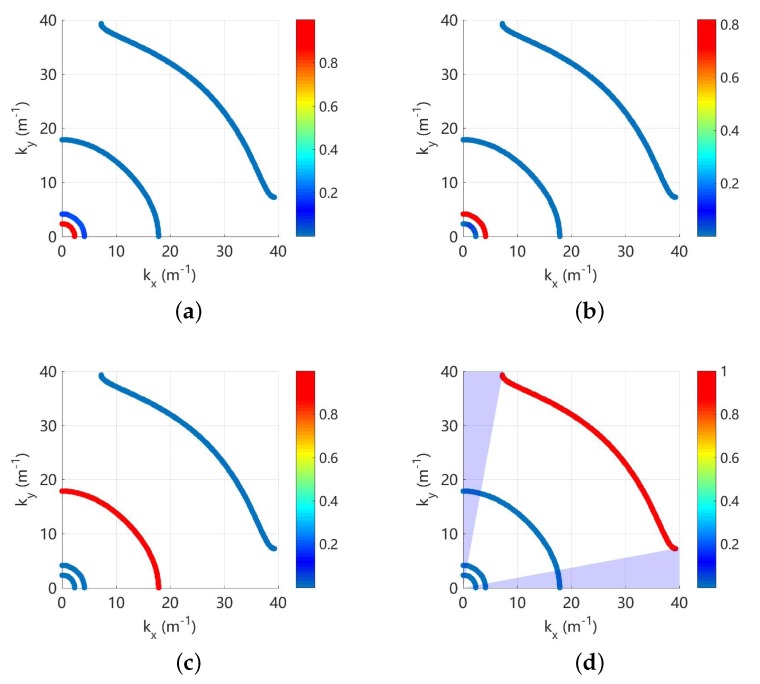
Wavenumber of the propagation waves at 800 Hz, coloured by: (**a**) ${\mathrm{RSW}}_{l}$; (**b**) ${\mathrm{RSW}}_{s}$; (**c**) ${\mathrm{RSW}}_{e}$; and (**d**) ${\mathrm{RSW}}_{f}$. The purple area in sub-figure (**d**) indicates the identified Bragg band gap for the flexural deformation.

As an example, these indicators are used to analyse the propagating waves at 800 Hz and the results are shown in Figure 8. There are very weak electromechanical coupling because the frequency is far away from the locking zone, so each wave contains one deformation. It can be seen that the longitudinal, shear, electric and flexural waves are clearly identified and match with the observation. The Bragg band gap for the flexural wave is also obtained, and we find it with strong directionality. It exists only in a small range of angles including zero, and this agrees with the results in Figure 6b.

**Figure 9:**
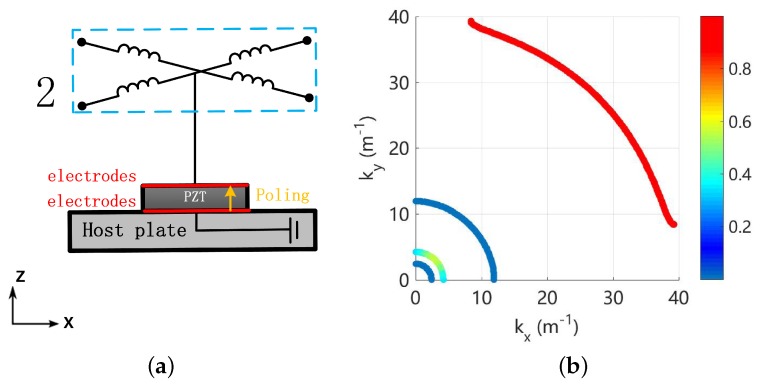
Wavenumber of the propagation waves (**b**) at 800 Hz for unit cell with single-sided PZT patch (**a**), coloured by ${\mathrm{RSW}}_{f}$.

To further explain the use of RSW, let us consider only bounding the PZT patches at one side of the plate, as shown in Figure 9a. The propagating waves at 800 Hz are analysed and shown in Figure 9b. The RSW results clearly indicate that there are two waves having significant contribution of the flexural deformation, and one of them can propagate along all directions. In this regard, there is no effective band gap for the flexural deformation, even though there is still a band gap for the wave with largest wavenumber. Note that in practice we often concern more about the band gap of a specific deformation that can be excited by the external forces, not the band gap of certain wave mode. These results also explain why we use collocated PZT patches: because we aim to get the effective band gaps for the flexural deformation.

### 3.3. Coupled Gap Versus Bragg Gap

**Figure 10:**
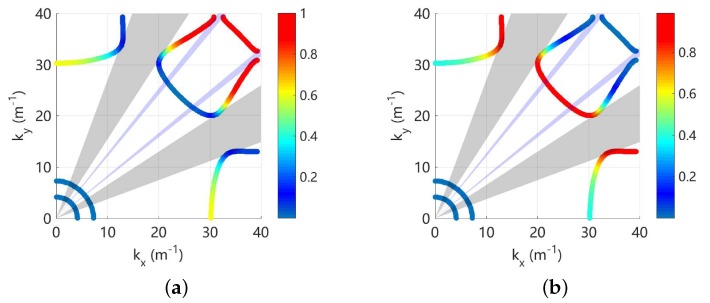
Wavenumber of the propagation waves at 1400 Hz for L-network with L=0.1 H: (**a**) coloured by ${\mathrm{RSW}}_{f}$ and (**b**) coloured by ${\mathrm{RSW}}_{e}$. The grey and purple areas indicate the coupled and Bragg gaps respectively.

Note that the Bragg band gaps co-exist with the coupled band gaps as shown in Figure 6, and the directionality of both band gaps at 1400 Hz is given in Figure 10. The propagation solutions $\left(k_{x},k_{y}\right)$ are coloured by the associated ${\mathrm{RSW}}_{f}$ or ${\mathrm{RSW}}_{e}$. Around the Bragg gap, ${\mathrm{RSW}}_{f}≃1$ and this indicates such a band gap is created by the evolution of flexural waves without any electromechanical coupling. On the contrary, both ${\mathrm{RSW}}_{f}$ and ${\mathrm{RSW}}_{e}$ are varying significantly near the coupled gaps. This confirms that the coupling gaps are generated by the interaction between two weakly coupled electromechanical waves.

**Figure 11:**
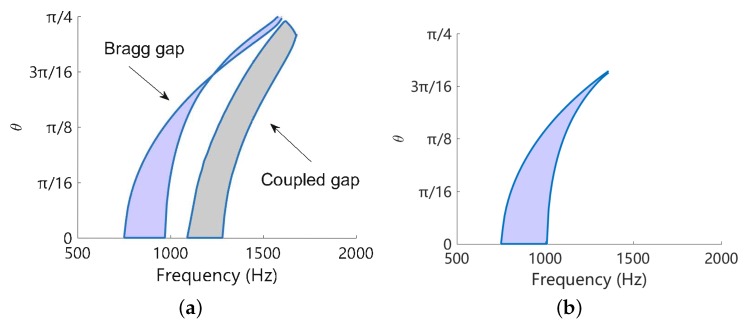
Variation of the angular width of band gaps with respect to the frequency: (**a**) L-network with L=0.1H; (**b**) without circuitry network.

From Figure 10 we can obtain the angular width of the band gaps. Redo this at frequency range [500,2000] Hz, the width variation of band gap for flexural deformation is obtained and shown in Figure 11a. Owing to the symmetry, we only present the results in $\left[0,\pi /4\right]$. We can observe that the bandwidth of the coupled band gap is approximately constant for most of the angles, except a small region around π/4. This is no longer hold for the Bragg band gap, for which the bandwidth varies dramatically with respect to the angle and becomes very narrow after π/8. This can be confirmed by the results shown in Figure 10. Similar conclusion can be drawn if the Bragg gap of the open-circuit case is used in the comparison, as shown in Figure 11b.

**Figure 12:**
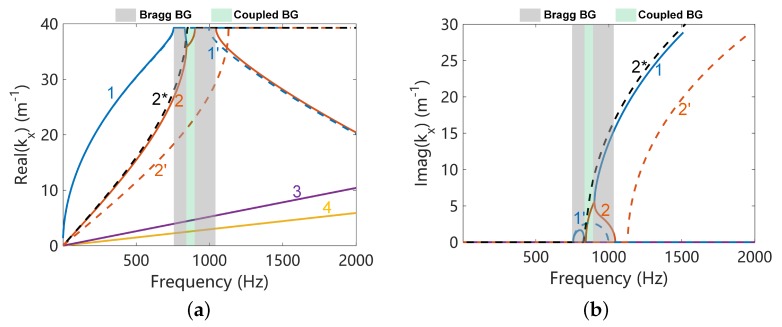
Dispersion curves along the *x* direction of L-network with L=0.2 H, (**a**) shows the real part of $k_{x}$ and (**b**) shows the imaginary part. Waves 1 and 2 are electromechanical waves. The dash lines 1’ and 2* refer to uncoupled disconnected flexural and electric waves respectively. The dash line 2’ refers to the uncoupled blocked electric wave.

**Figure 13:**
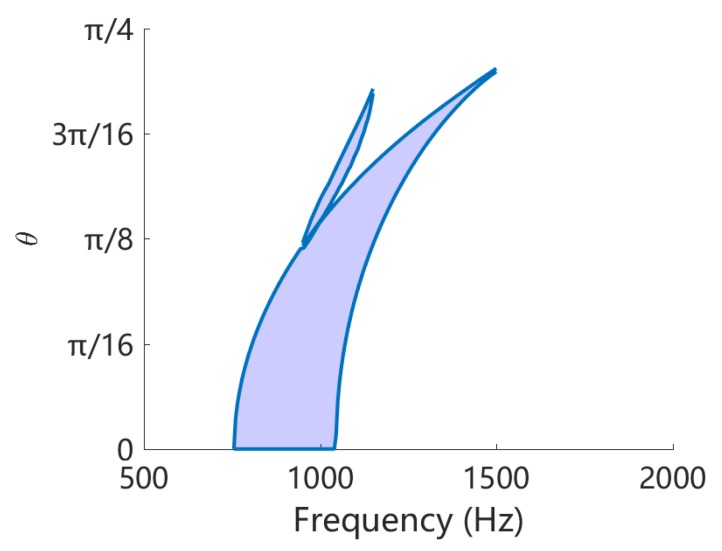
Variation of the angular width of band gaps with respect to the frequency, for L-network with L=0.2 H.

Increasing the network inductance to L=0.2 H, we found that the uncoupled electric wave intersects with the uncoupled mechanical wave in a Bragg band gap, where the group velocity of the mechanical wave is zero as shown in Figure 5. The coupled waves are shown in Figure 12. Interestingly, a coupled band gap still spears but inside the Bragg band gap, justifying by the frequencies in the Bragg gap where real(k)≠π. The attenuation (the imaginary part of *k*) is slightly enhanced, but both the angular and frequency width (Figure 13) are not extended significantly in comparison with original Bragg band gap as shown in Figure 11c. The uncoupled blocked electric wave is also presented, leading to an inaccurate prediction of the results. This once again justifies the use of the uncoupled disconnected waves in this paper.

**Figure 14:**
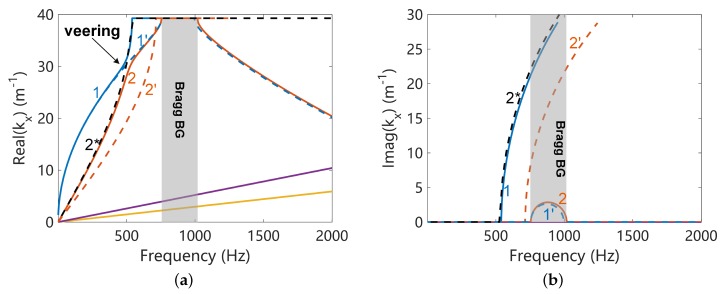
Dispersion curves along the *x* direction of L-network with L=0.5 H, (**a**) shows the real part of $k_{x}$ and (**b**) shows the imaginary part of $k_{x}$. Waves 1 and 2 are electromechanical waves; 3 and 4 are respectively shear wave and longitudinal wave. The dash lines 1’ and 2* refer to uncoupled disconnected flexural and electric waves respectively. The dash line 2’ refers to the uncoupled blocked electric wave.

**Figure 15:**
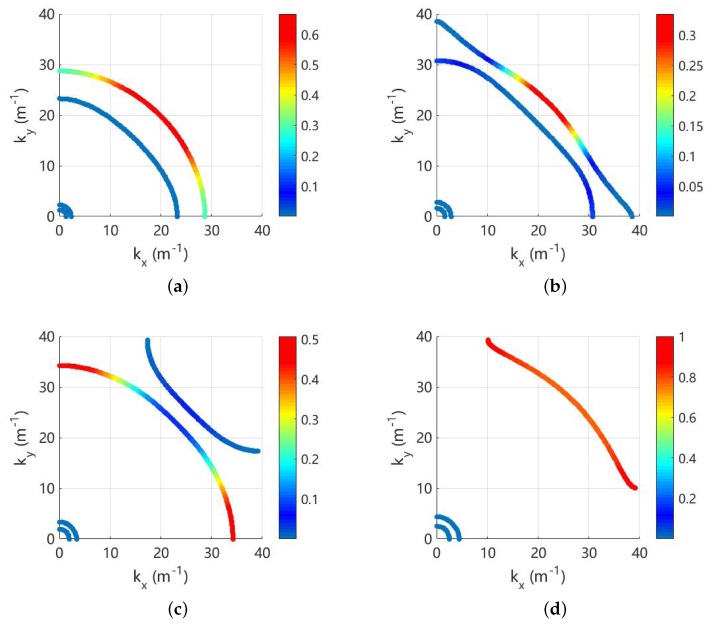
Wavenumber of the propagation waves for L-network with L=0.5 H, coloured by ${\mathrm{RSW}}_{f}$: (**a**) at 440 Hz; (**b**) at 540 Hz; (**c**) at 640 Hz; (**d**) at 840 Hz.

If we continue to increase the network inductance to L=0.5 H, the veering phenomenon rather than the locking between flexural wave and electric wave will happen, as shown in Figure 14. The veering zone is well predicted by the intersection of the uncoupled disconnected waves (1’ and 2*). We also confirm that the use of the uncoupled blocked wave (2’) is inappropriate. The ${\mathrm{RSW}}_{f}$ of the propagation waves at four representative frequencies are shown in Figure 15. When the frequency is low and far from the veering zone, i.e., at 440 Hz shown in Figure 15a, the wave with higher wavenumber is the one dominated by the flexural deformation. This wave is progressively coupled with the electric field when the frequency is approaching to the veering zone, as shown in Figure 15b. Interestingly the wave with lower wavenumber is dominated by the flexural deformation when the frequency is higher and two waves are separating, as shown in Figure 15c. This is termed ‘mode switching’ in the literature [17]. Note that the distribution of flexural wave is no longer a quarter of circle arc in the veering zone, indicating that it no longer propagates uniformly. In Figure 15d there are only 3 propagation waves because the wave dominated by the electric field is in a permanent band gap.

Although the veering phenomenon cannot be used to generate a coupled band gap, it does facilitate strong energy exchange between the electric and mechanical field. This may lead to significant damping when resistance is introduced. Moreover, it makes the flexural deformation can only be transmitted via a limited angles, as shown in Figure 15b,c. All these aspects can be used to control the vibration and energy transmission.

### 3.4. Coupled Gap Versus LR Gap

In the previous sections, we showed that the coupled band gaps can be created by an electric network with only inductance (L-network). It may be interesting to compare the features of the coupled band gap with the local resonance (LR) gap, because the later is also created only by inductance through local impedance (L-shunt). Especially, it is worthy to conduct the comparison under the same overall inductance.

**Figure 16:**
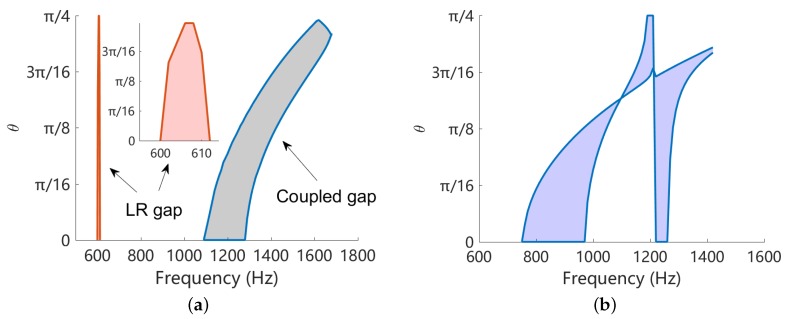
(**a**) Comparison of coupled band gaps created by L-network with L=0.1 H and LR band gap created by L-shunt with L=0.4 H. The overall inductance of of such two cases are the same; (**b**) Variation of the angular width of band gaps with respect to the frequency, for L-shunt with L=0.05 H. In this case the LR band gap is merged with the Bragg band gap.

The comparison is summarized in Figure 16a, where the coupled band gap is created by L-network with L=0.1 H and the LR band gap created by L-shunt with L=0.4 H. The overall inductance of such two cases are the same, because each unit cell of L-network consists of 4 inductors. We can see that the LR band gap is an absolute band gap that forbids the flexural wave to propagate along any direction. The coupled band gap also covers all the angles but the frequency zone changes with the angle. This is so, because currently we are using square piezoelectric patches, and this exhibits different periodicity for different angles. We might use circular patches and design the periodic pattern to mitigate this tendency and achieve a nearly absolute band gap. On the other hand, the LR gap is quite narrow (less than 10 Hz) comparing to the coupled band gap (approximately 200 Hz). This is the main advantage of the coupled band gap.

Note that if we intentionally design the LR gap such that it is merged with the Bragg gap, a ‘super’ band gap can be created [49], as shown in Figure 16b. Despite complex, this does cover a wider space in the θ-frequency plane. Recall the situation when we attempted to merge the coupled band gap with the Bragg band gap, shown in Figure 13, the overall band gap is not significantly intensified. It may be interesting to further study the interaction of the coupled band gap with other types of band gaps.

## 4. Validation of the Coupled Band Gaps

**Figure 17:**
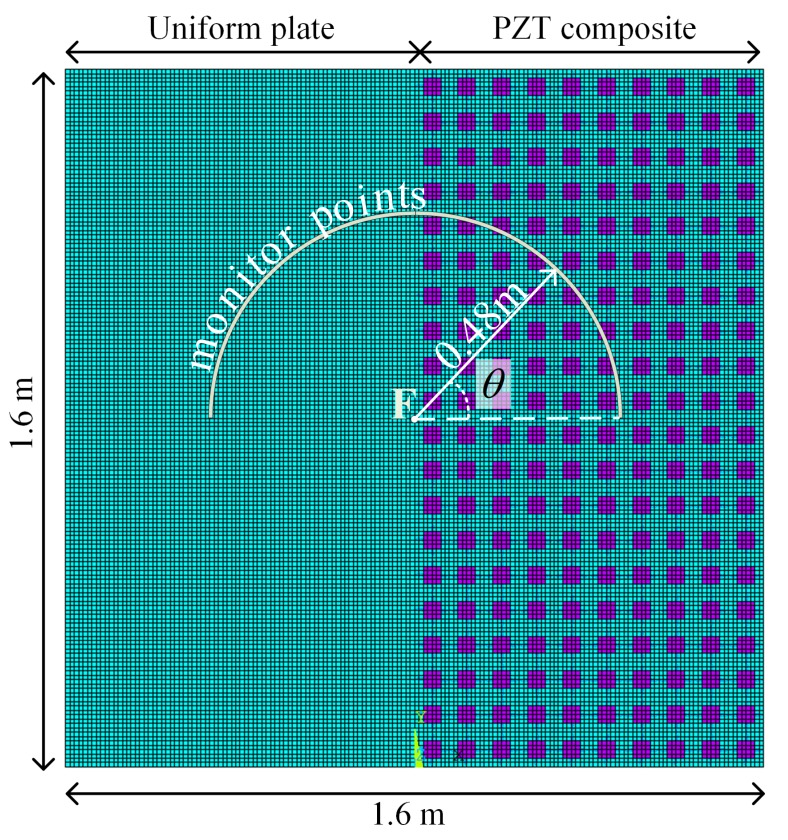
Finite element model used for validation purpose.

In this section, we conduct a numerical analysis concerning the transient forced response on an intentionally designed plate, so as to verify the results presented in Section 3.2. The analysed structure is shown in Figure 17 where the piezoelectric system is only implemented on the right half of the host plate. The plate has finite extent and free boundary conditions are applied to its four sides. The unit cell of the PZT composite is the same as shown in Figure 4a. A network impedance is imposed with only inductance L=0.1H between two adjacent unit cells. Such a piezoelectric composite is analysed in the previous sections: the dispersion curves along x direction are presented in Figure 6; the angular bandwidth of the Bragg and coupled band gap at different frequencies are presented in Figure 11. The dynamics of the piezoelectric composite can be validated by comparing the results with the right part of the structure.

A transient force is applied to the centre of the plate, namely the ‘F’ point in Figure 17. To minimize dispersion effects, narrow band signals are used, composed of 3 cycles modulated by a Hanning window with the central frequency $f_{0}$ equals to 700 Hz, 900 Hz and 1300 Hz respectively. The maximum amplitude is 0.5 N and the sampling frequency is 30 times greater than the central frequency in order to guarantee the signal quality of the wave packet. The initial displacement, velocity and accelerations all equal to zero. We do not consider any forms of damping (or resistive impedance) in the calculation. The forced response is calculated by ANSYS 17 using the ‘FULL’ option, which means that no model reduction techniques are applied. We use this option to ensure the accuracy of the results.

To monitor the angular energy propagation characteristics, a set of elements is selected at the arc whose radius is six times the unit cell, namely 0.48 m, as illustrated in Figure 17. For the piezoelectric composite, the propagation angle θ=0 along the positive x direction. For the uniform (half) plate, the propagation angle θ=0 along the negative x direction for the sake of comparison.

**Figure 18:**
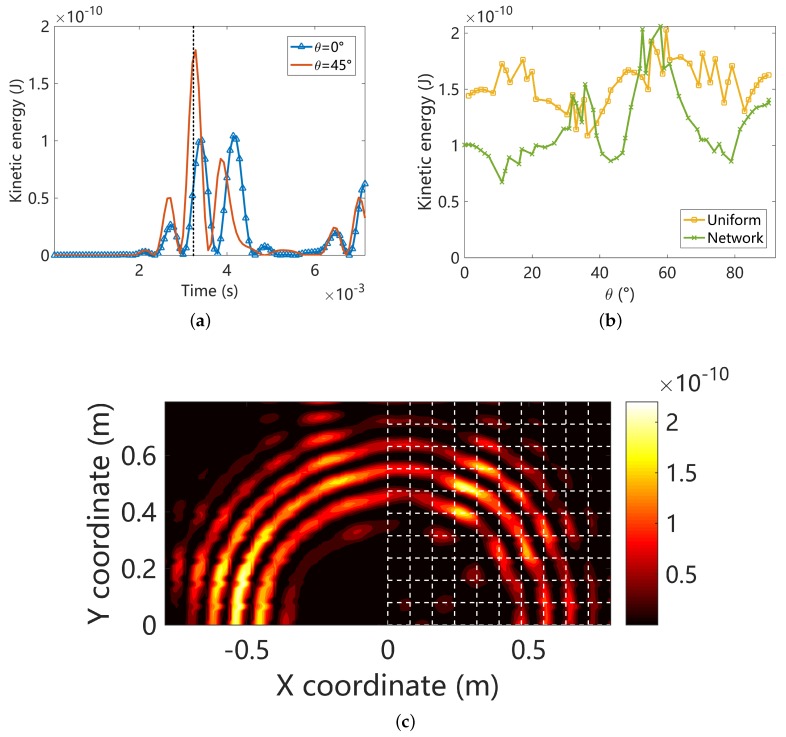
Summaries of the forced response results at 700 Hz: (**a**) the time-history of the element kinetic energies at the monitor points with propagation angle θ=0 and θ=π/4 in the piezoelectric composite; (**b**) The angular distribution of the instantaneous element kinetic energy at the monitor points; (**c**) The distribution of the instantaneous kinetic energy of the whole structure (only the upper half is shown due to symmetry). The results in Figure 18b,c are obtained at the same time point $t=3.4\times 10^{-3}\;s$ marked by the dotted line in Figure 18a.

First, let us set the central frequency of the wave package to 700 Hz, and the forced response are summarized in Figure 18. Frequency 700 Hz locates at the first propagating zone along arbitrary angle according to Figure 11. This implies that the energy can be delivered by the propagating waves along all the angles in the piezoelectric composite, and the point is verified by Figure 18c. However the kinetic energy does not distribute perfectly uniform along different angles, as shown in Figure 18a. This happens even for the uniform (half) plate, as shown in Figure 18b. For the uniform plate, this might be induced by scattering of energy from the border between the uniform and composite plates. For the piezoelectric composite, the dispersion relations are naturally different along different angles, despite that at lower frequencies it can be regarded as a homogeneous media. For these reasons, the non-uniform of the kinetic energy is observed.

**Figure 19:**
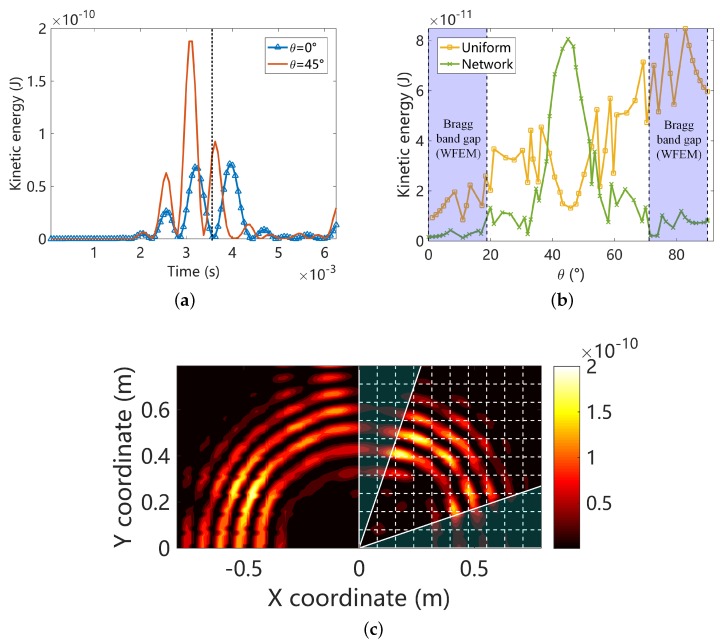
Summaries of the forced response results at 900 Hz: (**a**) the time-history of the element kinetic energies at the monitor points with propagation angle θ=0 and θ=π/4 in the piezoelectric composite; (**b**) The angular distribution of the instantaneous element kinetic energy at the monitor points; (**c**) The distribution of the instantaneous kinetic energy of the whole structure (only the upper half is shown due to symmetry). In Figure 19b,c, the results are obtained at the same time point $t=3.6\times 10^{-3}\;s$ marked by the dotted line in Figure 19a, and the identified band gap angles are highlighted by the blue areas.

Second, we set the central frequency of the wave package to 900 Hz, which locates inside the a Bragg band gap along some angles as shown in Figure 11. The forced response is summarized in Figure 19. The kinetic energy difference between monitor points along θ=0 and θ=π/4 is enlarged in comparison with Figure 18a. This is so, because 900 Hz is inside the band gap along θ=0 but in the propagation zone along θ=π/4. It can be observed in Figure 19b that the kinetic energy distribution along different angles in the uniform plate is highly non-uniform. Note that the 1/2 of uniform plate is connected to the piezoelectric composite plate from the left side and connected to another 1/2 uniform plate, as shown in Figure 17. At 900 Hz, band gaps emerge along some angles therefore the piezoelectric composite no longer behaves like a homogeneous media. For these reasons the results for the uniform plate in Figure 19b is highly asymmetric with π/4. However, the results for the piezoelectric composite in Figure 19b is approximately symmetric with π/4 and the energy is concentrated around π/4. This can be attributed to the weak energy transfer capability of the evanescent waves. The boundary conditions become less influential the structural dynamics inside a Bragg band gap [6]. The low kinetic areas match very with the band gap angles identified by RSW as shown in Figure 19b and more intuitively in Figure 19c.

**Figure 20:**
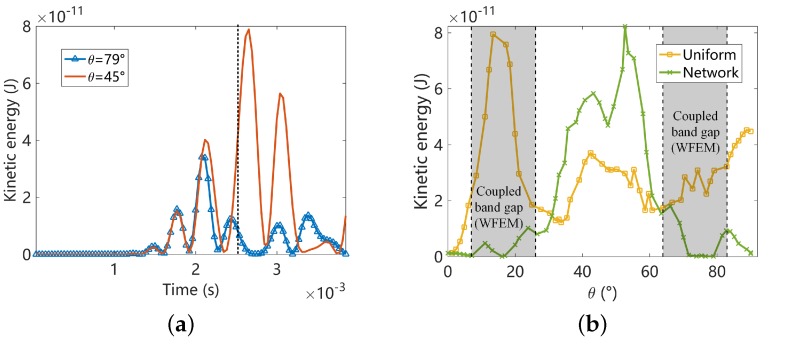

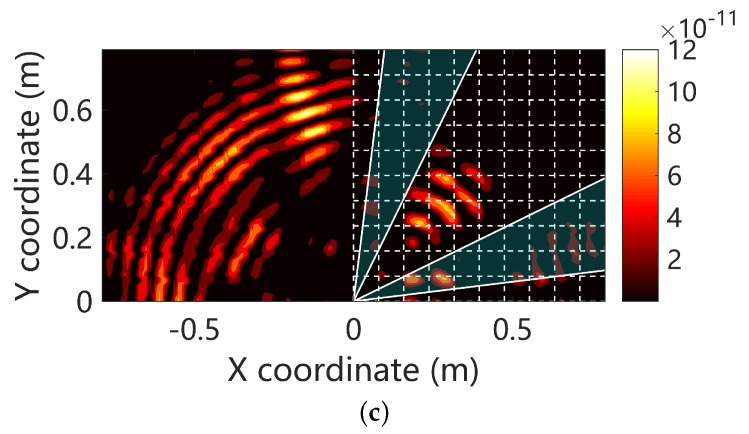
Summaries of the forced response results at 1300 Hz: (**a**) the time-history of the element kinetic energies at the monitor points with propagation angle $\theta =79{}^{\circ }$ and θ=π/4 in the piezoelectric composite; (**b**) The angular distribution of the instantaneous element kinetic energy at the monitor points; (**c**) The distribution of the instantaneous kinetic energy of the whole structure (only the upper half is shown due to symmetry). In Figure 20b,c, the results are obtained at the same time point $t=2.6\times 10^{-3}\;s$ marked by the dotted line in Figure 20a, and the identified band gap angles are highlighted by the grey areas.

**Figure 21:**
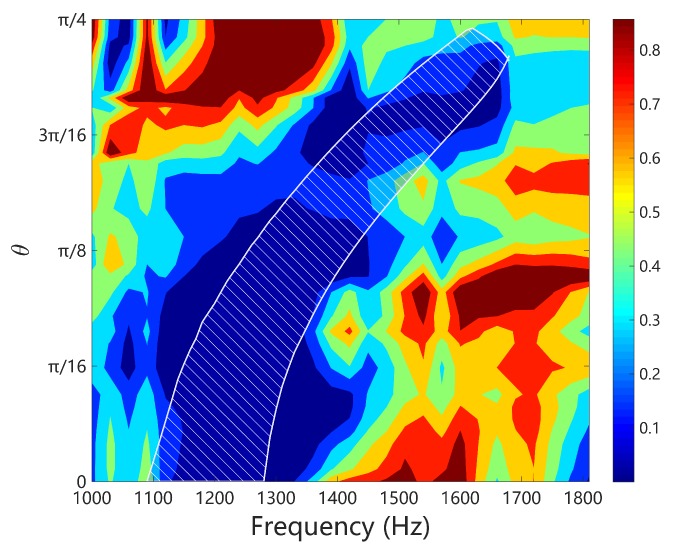
The angular distribution of the kinetic energy (normalized in percentage) at the monitoring points from 1000 Hz to 1800 Hz. The while patched area indicates the angular frequency location of the coupled band gap predicted by WFEM and identified by RSW, as shown in Figure 11.

At last, we set the central frequency of the wave package to 1300 Hz, which locates inside the a coupled band gap along some angles as shown in Figure 11. The forced response is summarized in Figure 20. We observe similar phenomenon, such as the energy concentration, the non-symmetric angular distribution of the kinetic energy for the uniform plate, and the near symmetric distribution for the piezoelectric composite in Figure 20a,b. The directionality of the energy flow is clearly presented in Figure 20c and matches very well with the results identified by RSW. Figure 21 compares the angular distribution of the kinetic energy (normalized in percentage) with the angular frequency location of the coupled band gap predicted by WFEM and identified by RSW. A good agreement between the low energy zone and the band gap is observed. Hence the existence of the coupled band gaps is completely verified. The results presented in this section also illustrate the potential application in vibration isolation using the directionality of the coupled band gap.

## 5. Conclusions

We show that a coupled band gap can be created by the locking phenomenon between two electromechanical waves in the piezoelectric composite plates. By designing the interconnected electric network, the uncoupled electric wave can be tailored such that the locking phenomenon can be triggered. Namely if the uncoupled disconnected electric and mechanical waves have opposite sign of group velocity when intersect, a coupled band gap will be created and centred on intersection frequency. Otherwise if they have the same sign of group velocity, the veering phenomenon will occur and there will be no such a coupled band gap.

Through piezoelectric effects, the uncoupled electric and mechanical waves are tailored into two electromechanical waves. At most of the frequencies, such two waves are dominated by electric or mechanical (flexural) fields. They exhibit strong coupling only at the locking (coupled band gap) and veering zones. An indicator is proposed to identify the effective band gap for a given deformation. It is applied to identify the band gaps for the flexural deformation in this paper.

The coupled band gap has similar bandwidth as the Bragg band gap in the best situation. However, the bandwidth of coupled band gap hardly vary with the propagation angle. This may be a favourable feature for the application of controlling the energy transmission.

The bandwidth of the coupled band gap is much larger than the LR band gap when the overall inductance is the same. Both band gaps cover all the propagation angle. The LR band gap does this at the same frequency zone, but the frequencies of the coupled band gap vary with propagation angle. In the considered case, the coupled band gap cannot cover all the angle for a same frequency. In future work, this may be improved by the geometric design of the PZT patches.

The wave finite element method can be used, in association with the proposed metrics termed ‘Ratio of Single Wave’ to predict and identify the coupled band gaps. The methods are verified by the analytical solutions and the forced response results with good agreements. The overall work in this paper shows a promising approach to adaptively generate the coupled band gaps, and this can find applications in vibration control, energy focusing and so on. Future work involves the design of the electric network to establish extensive coupled band gaps and experimental studies.

## Abbreviations

M	generalized mass matrix
C	generalized damping matrix
K	generalized stiffness matrix
q	generalized nodal displacement vector
f	generalized force vector
D, $\hat{D}$	dynamic stiffness matrix
I	identity matrix
X, Y, Z	matrices
μ	propagation constants
*k*	wavenumber, $m^{-1}$
*E*	Young’s modulus, $N/m^{2}$
ν	Poisson’s ratio, dimensionless
ρ	mass density, $\mathrm{kg}/m^{3}$
*h*	thickness, m
ω	circular frequency, rad/s
θ	rotation angle
λ	eigenvalue
*L*	inductance, H or length, m
*t*	time, s
Superscripts:	
T	matrix transpose
H	conjugate transpose
Subscripts:	
*x*,*y*,*z*	quantity/property pertaining in the respective direction
b	quantity related to the boundary nodes
i	quantity related to the internal nodes
1,2,3,4	quantity related to corner nodes
L,B,R,T	quantity related to left, bottom, right, top ends of the unit cell
E	quantity related to the electric field
M	quantity related to the mechanical field
l	quantity related to the longitudinal wave
s	quantity related to the shear wave
f	quantity related to the flexural wave
e	quantity related to the electric wave
ref	quantity related to the reference wave shape
full	quantity related to the actual wave shape

## Appendix A. Formulation of the spectral eigenvalue problem (12)

According to Equations (5) and (6), Equation (4) is rewritten as follows:

(A1) $$\left[\begin{array}{cccccccc} D_{11} & D_{12} & D_{13} & D_{14} & D_{1L} & D_{1B} & D_{1R} & D_{1T}\\ D_{21} & D_{22} & D_{23} & D_{24} & D_{2L} & D_{2B} & D_{2R} & D_{2T}\\ D_{31} & D_{32} & D_{33} & D_{34} & D_{3L} & D_{3B} & D_{3R} & D_{3T}\\ D_{41} & D_{42} & D_{43} & D_{44} & D_{4L} & D_{4B} & D_{4R} & D_{4T}\\ D_{L1} & D_{L2} & D_{L3} & D_{L4} & D_{\mathrm{LL}} & D_{\mathrm{LB}} & D_{\mathrm{LR}} & D_{\mathrm{LT}}\\ D_{B1} & D_{B2} & D_{B3} & D_{B4} & D_{\mathrm{BL}} & D_{\mathrm{BB}} & D_{\mathrm{BR}} & D_{\mathrm{BT}}\\ D_{R1} & D_{R2} & D_{R3} & D_{R4} & D_{\mathrm{RL}} & D_{\mathrm{RB}} & D_{\mathrm{RR}} & D_{\mathrm{RT}}\\ D_{T1} & D_{T2} & D_{T3} & D_{T4} & D_{\mathrm{TL}} & D_{\mathrm{TB}} & D_{\mathrm{TR}} & D_{\mathrm{TT}} \end{array}\right]\left(\begin{array}{c} q_{1}\\ q_{2}\\ q_{3}\\ q_{4}\\ q_{L}\\ q_{B}\\ q_{R}\\ q_{T} \end{array}\right)=\left(\begin{array}{c} f_{1}\\ f_{2}\\ f_{3}\\ f_{4}\\ f_{L}\\ f_{B}\\ f_{R}\\ f_{T} \end{array}\right)$$

and according to Equations (8) and (11), the eigenvalue problem (12) can be rewritten as:

(A2) $$\left(\lambda_{x}\left[\begin{array}{ccc} X_{11} & X_{1L} & X_{1B}\\ X_{L1} & X_{\mathrm{LL}} & X_{\mathrm{LB}}\\ X_{B1} & X_{\mathrm{BL}} & X_{\mathrm{BB}} \end{array}\right]+\left[\begin{array}{ccc} Y_{11} & Y_{1L} & Y_{1B}\\ Y_{L1} & Y_{\mathrm{LL}} & Y_{\mathrm{LB}}\\ Y_{B1} & Y_{\mathrm{BL}} & Y_{\mathrm{BB}} \end{array}\right]+\lambda_{x}^{-1}\left[\begin{array}{ccc} Z_{11} & Z_{1L} & Z_{1B}\\ Z_{L1} & Z_{\mathrm{LL}} & Z_{\mathrm{LB}}\\ Z_{B1} & Z_{\mathrm{BL}} & Z_{\mathrm{BB}} \end{array}\right]\right)\left(\begin{array}{c} q_{1}\\ q_{L}\\ q_{B} \end{array}\right)=0$$

where

(A3) $$X_{11}=D_{12}+D_{34}+D_{32}\lambda_{y}^{-1}+D_{14}\lambda_{y}$$

(A4) $$X_{1L}=D_{1R}+D_{3R}\lambda_{y}^{-1}$$

(A5) $$X_{L1}=D_{L2}+D_{L4}\lambda_{y}$$

(A6) $$X_{\mathrm{LL}}=D_{\mathrm{LR}}$$

(A7) $$X_{B1}=D_{B2}+D_{T4}+D_{T2}\lambda_{y}^{-1}+D_{B4}\lambda_{y}$$

(A8) $$X_{\mathrm{BL}}=D_{\mathrm{BR}}+D_{\mathrm{TR}}\lambda_{y}^{-1}$$

(A9) $$X_{1B}=X_{\mathrm{LB}}=X_{\mathrm{BB}}=0$$

(A10) $$Y_{11}=D_{11}+D_{22}+D_{33}+D_{44}+\left(D_{31}+D_{42}\right)\lambda_{y}^{-1}+\left(D_{13}+D_{24}\right)\lambda_{y}$$

(A11) $$Y_{1L}=D_{1L}+D_{2R}+\left(D_{3L}+D_{4R}\right)\lambda_{y}^{-1}$$

(A12) $$Y_{1B}=D_{1B}+D_{3T}+D_{3B}\lambda_{y}^{-1}+D_{1T}\lambda_{y}$$

(A13) $$Y_{L1}=D_{L1}+D_{R2}+\left(D_{L3}+D_{R4}\right)\lambda_{y}$$

(A14) $$Y_{\mathrm{LL}}=D_{\mathrm{LL}}+D_{\mathrm{RR}}$$

(A15) $$Y_{\mathrm{LB}}=D_{\mathrm{LB}}+D_{\mathrm{LT}}\lambda_{y}$$

(A16) $$Y_{B1}=D_{B1}+D_{T3}+D_{T1}\lambda_{y}^{-1}+D_{B3}\lambda_{y}$$

(A17) $$Y_{\mathrm{BL}}=D_{\mathrm{BL}}+D_{\mathrm{TL}}\lambda_{y}^{-1}$$

(A18) $$Y_{\mathrm{BB}}=D_{\mathrm{BB}}+D_{\mathrm{TT}}+D_{\mathrm{TB}}\lambda_{y}^{-1}+D_{\mathrm{BT}}\lambda_{y}$$

(A19) $$Z_{11}=D_{21}+D_{43}+D_{41}\lambda_{y}^{-1}+D_{23}\lambda_{y}$$

(A20) $$Z_{1L}=D_{2L}+D_{4L}\lambda_{y}^{-1}$$

(A21) $$Z_{1B}=D_{2B}+D_{4T}+D_{4B}\lambda_{y}^{-1}+D_{2T}\lambda_{y}$$

(A22) $$Z_{L1}=D_{R1}+D_{R3}\lambda_{y}$$

(A23) $$Z_{\mathrm{LL}}=D_{\mathrm{RL}}$$

(A24) $$Z_{\mathrm{LB}}=D_{\mathrm{RB}}+D_{\mathrm{RT}}\lambda_{y}$$

(A25) $$Z_{B1}=Z_{\mathrm{BL}}=Z_{\mathrm{BB}}=0$$

## Appendix B. Material properties of PZT-5H

Mass density: ρ=7500 kg/m${}^{3}$.

Material stiffness matrix evaluated at constant electric field:

$$c^{E}=10^{10}\times \left[\begin{array}{cccccc} 12.7 & 8.02 & 8.47 & & & \\ 8.02 & 12.7 & 8.47 & & & \\ 8.47 & 8.47 & 11.7 & & & \\ & & & 2.3 & & \\ & & & & 2.3 & \\ & & & & & 2.35 \end{array}\right]\mathrm{Pa}$$

Permittivity matrix evaluated at constant strain:

$$\varepsilon^{S}=\varepsilon_{0}\times \left[\begin{array}{ccc} 1703.7 & & \\ & 1703.7 & \\ & & 1432.7 \end{array}\right]$$

where $\varepsilon_{0}=8.85\times 10^{-12}$ C/(V·m).

Piezoelectric stress coupling matrix:

$$e=\left[\begin{array}{ccc} & & -6.5\\ & & -6.5\\ & & 23.3\\ & 0 & \\ & 17 & \\ 17 & & \end{array}\right]N/\left(V\;\cdot \;m\right)$$

## Appendix C. Theoretical solutions for the uncoupled disconnected electric waves

When analysing the wave propagation along the x direction, we set $\lambda_{y}=1$ and searching for $\lambda_{x}$ at each frequency ω. In this way, the dynamic equation of the unit cell shown in Figure 4b writes:

(A26) $$\left[\begin{array}{cc} \frac{1}{L} & -\frac{1}{L}\\ -\frac{1}{L} & \frac{1}{L}-\omega^{2}C \end{array}\right]\left(\begin{array}{c} q_{L}\\ q_{R} \end{array}\right)=\left(\begin{array}{c} f_{L}\\ f_{R} \end{array}\right)$$

where *q* refers to the magnetic flux (whose first derivative with time is voltage), and *f* refers to the charge (whose first derivative with time is current), the subscripts follow the same meanings as mentioned in Section 2. Please note that $q_{B}$ and $q_{T}$ are omitted for $\lambda_{y}=1$, and the equation is written in the context of harmonic motion. Imposing the periodic boundary conditions $f_{R}=\lambda_{x}f_{L}$ and $f_{L}+\lambda_{x}^{-1}f_{R}=0$, we have:

(A27) $$\left[\begin{array}{cc} 1 & \lambda_{x}^{-1} \end{array}\right]\left[\begin{array}{cc} \frac{1}{L} & -\frac{1}{L}\\ -\frac{1}{L} & \frac{1}{L}-\omega^{2}C \end{array}\right]\left[\begin{array}{c} 1\\ \lambda_{x} \end{array}\right]q_{L}=0$$

leading to the solving of the following polynomial equation concerning $\lambda_{x}$:

(A28) $$\frac{1}{\lambda_{x}L}\left(\lambda_{x}^{2}+\left(\omega^{2}CL-2\right)\lambda_{x}+1\right)=0$$

where

(A29) $$\lambda_{x}=e^{-jk_{x}L_{x}}$$

Finally, we obtain that

(A30) $$k_{x}=j\left[\ln\left(2-\omega^{2}CL\pm \sqrt{{\left(\omega^{2}CL\right)}^{2}-4\omega^{2}CL}\right)-\ln\left(2\right)\right]/L_{x}$$

Giving a frequency ω, the wavenumber $k_{x}$ can be obtained. Please noted that $C=3.5714\times 10^{-7}\;H$ for the uncoupled disconnected wave, and $C=1.9824\times 10^{-7}\;H$ for the uncoupled blocked wave of considered piezoelectric plates.
